# TGF-β downstream of Smad3 and MAPK signaling antagonistically regulate the viability and partial epithelial–mesenchymal transition of liver progenitor cells

**DOI:** 10.18632/aging.205725

**Published:** 2024-04-05

**Authors:** Yi-Min Sun, Yu Wu, Gan-Xun Li, Hui-Fang Liang, Tu-Ying Yong, Zifu Li, Bixiang Zhang, Xiao-Ping Chen, Guan-Nan Jin, Ze-Yang Ding

**Affiliations:** 1 Hepatic Surgery Center, Hubei Province for The Clinical Medicine Research Center of Hepatic Surgery and Hubei Key Laboratory of Hepatic-Biliary-Pancreatic Diseases, Tongji Hospital, Tongji Medical College, Huazhong University of Science and Technology, Wuhan, Hubei 430030, China; 2 Present address: Department of Gastrointestinal Surgery, Affiliated First Hospital, Yangtze University, Jingzhou, Hubei 434000, China; 3 National Engineering Research Center for Nanomedicine, College of Life Science and Technology, Huazhong University of Science and Technology, Wuhan, Hubei 430071, China; 4 Present address: Department of Nephrology, Union Hospital, Tongji Medical College, Huazhong University of Science and Technology, Wuhan, Hubei 430030, China

**Keywords:** liver progenitor cells, proliferation, partial epithelial–mesenchymal transition, TGF-β, Smad3, MAPK signaling

## Abstract

Background: Liver progenitor cells (LPCs) are a subpopulation of cells that contribute to liver regeneration, fibrosis and liver cancer initiation under different circumstances.

Results: By performing adenoviral-mediated transfection, CCK-8 analyses, F-actin staining, transwell analyses, luciferase reporter analyses and Western blotting, we observed that TGF-β promoted cytostasis and partial epithelial–mesenchymal transition (EMT) in LPCs. In addition, we confirmed that TGF-β activated the Smad and MAPK pathways, including the Erk, JNK and p38 MAPK signaling pathways, and revealed that TGFβ-Smad signaling induced growth inhibition and partial EMT, whereas TGFβ-MAPK signaling had the opposite effects on LPCs. We further found that the activity of Smad and MAPK signaling downstream of TGF-β was mutually restricted in LPCs. Mechanistically, we found that TGF-β activated Smad signaling through serine phosphorylation of both the C-terminal and linker regions of Smad2 and 3 in LPCs. Additionally, TGFβ-MAPK signaling inhibited the phosphorylation of Smad3 but not Smad2 at the C-terminus, and it reinforced the linker phosphorylation of Smad3 at T179 and S213. We then found that overexpression of mutated Smad3 at linker phosphorylation sites intensifies TGF-β-induced cytostasis and EMT, mimicking the effects of MAPK inhibition in LPCs, whereas mutation of Smad3 at the C-terminus caused LPCs to blunt TGF-β-induced cytostasis and partial EMT.

Conclusion: These results suggested that TGF-β downstream of Smad3 and MAPK signaling were mutually antagonistic in regulating the viability and partial EMT of LPCs. This antagonism may help LPCs overcome the cytostatic effect of TGF-β under fibrotic conditions and maintain partial EMT and progenitor phenotypes.

## INTRODUCTION

Liver progenitor cells (LPCs) are a population of cells with characteristics of ovoid nuclei and the potential to differentiate into hepatocytes and cholangiocytes [1]. LPCs usually arise and expand in the canal of Hering of the injured liver and contribute to liver regeneration and repair [2]. However, we and others have confirmed that under pathological circumstances, LPCs respond to fibrotic or carcinogenic cytokines or regimens [3, 4] and participate in liver fibrosis and liver cancer initiation through the production of extracellular matrix (ECM), epithelial–mesenchymal transition (EMT), and malignant transformation [5–7]. In the context of liver injury, which includes viral hepatitis, fatty liver disease, autoimmune hepatitis, and cirrhosis, transforming growth factor-β (TGF-β) is one of the most common fibrotic cytokines [8]. In detail, TGF-β phosphorylates and activates the SMAD2/3 complex through the TGF-β receptor, and phosphorylated SMAD2/3 translocates into the nucleus to induce the expression of fibrotic or carcinogenic genes, such as connective tissue growth factor (CTGF) and c-Jun. We and others have revealed that in LPCs, TGF-β induces cytostasis, the production of ECM components, such as cCTGF, EMT, and malignant transformation [4, 9–11]. Moreover, our group reported that autocrine TGF-β signaling occurs and induces partial EMT in LPCs [12]. However, the exact mechanism, crosstalk, and related mechanism of action of TGF-β downstream signaling in LPCs have not been explored.

In this study, we demonstrated that TGF-β induced limited cytostasis and EMT and that TGF-β-activated Smad and non-Smad mitogen-activated protein kinase (MAPK) signaling regulated these effects in LPCs. Interestingly, we discovered that TGF-β downstream of Smad3 and MAPK signaling has antagonistic effects on the TGF-β-induced growth inhibition and EMT of LPCs.

## RESULTS

### TGF-β induced growth inhibition and partial epithelial–mesenchymal transition in liver progenitor cells

**Figure 1:**
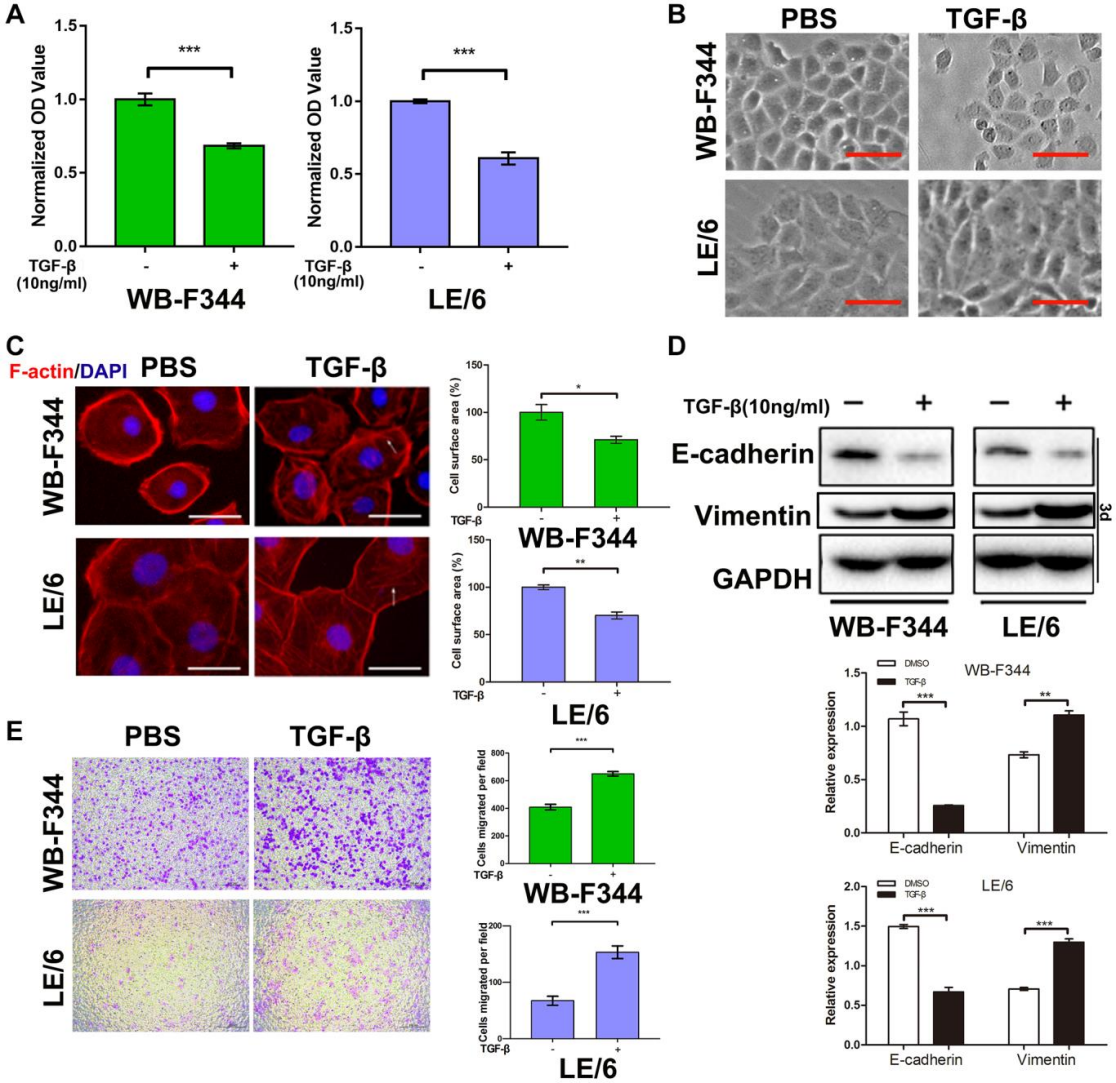
**TGF-β inhibited the growth and promoted epithelial–mesenchymal transition in liver progenitor cells.** (**A**) Liver progenitor cell lines (WB-F344 and LE/6) were treated with TGF-β (10 ng/ml) for 3 days and then subjected to CCK-8 analyses. OD values were normalized to those of the control groups. (**B**) Phase contrast images of TGF-β (10 ng/ml, 3 days)-treated WB-F344 and LE/6 cells. Scale bar, 100 μm. (**C**) Phalloidin staining for F-actin (red) in TGF-β (10 ng/ml, 3 days)-induced WB-F344 and LE/6 cells. DAPI was used to show the location of the nucleus (blue). The white arrows indicate rearrangements of F-actin. Scale bar, 25 μm. (**D**) Western blot analyses of the indicated cell lines with antibodies against E-cadherin and vimentin. GAPDH was used as a loading control. (**E**) Transwell analyses of the motility of WB-F344 and LE/6 cells treated with or without TGF-β (10 ng/ml, 7 hr and 24 hr). Representative images of migrated cells are shown (left panel); migrated cells were counted, and the average number of cells in three independent experiments is shown (right panel). Two-tailed Student’s *t*-tests were used for statistical analysis. The data are presented as the mean ± S.E.M. ^***^p < 0.001.

To investigate the role of TGF-β and its downstream signaling in liver progenitor cells, we first treated liver progenitor cell lines (WB-F344 and LE/6 cells) with TGF-β (10 ng/ml) for 3 days and then analyzed cell proliferation (CCK-8) assays (repeated three times). After treatment with TGF-β, the proliferation of WB-F344 and LE/6 cells was reduced (Figure 1A), although the inhibitory effect on cell growth was limited to both WB-F344 (39.24% inhibition, *P* < 0.001 for the TGF-β-treated vs. the PBS-treated WB-F344 cell line) and LE/6 (31.67% inhibition, *P* < 0.0001 for the TGF-β-treated vs. the PBS-treated LE/6 cell line) cells. Considering that TGF-β is a classic inducer of epithelial–mesenchymal transition (EMT) in cells and that EMT and partial EMT of LPCs contribute to liver fibrosis and the initiation of liver cancer [12–14], we subsequently performed EMT-related experiments to confirm whether EMT occurs in LPCs after treatment with TGF-β. Intriguingly, after exposure to a high concentration of TGF-β (10 ng/ml) for 3 days, phase contrast microscopic scanning and fluorescent F-actin staining revealed morphological changes (Figure 1B) and rearrangement of actin fibers (Figure 1C) in both WB-F344 and LE/6 cells, although this trend was not obvious. Western blot analyses of WB-F344 and LE/6 cells after treatment with TGF-β (10 ng/ml) for three days revealed that the expression of an epithelial marker (E-cadherin) was reduced, while the expression of a mesenchymal marker (vimentin) was slightly increased (Figure 1D and Supplementary Figure 1). *In vitro* motility assays showed that the migration of WB-F344 and LE/6 cells was enhanced by stimulation with TGF-β (Figure 1E), which suggests that the mesenchymal state of LPCs was enhanced after stimulation with TGF-β. Taken together, these results suggested that despite TGF-β-induced cytostasis and EMT in LPCs, these effects were not obvious.

### Both Smad and MAPK signaling were activated in liver progenitor cells under TGF-β stimulation

**Figure 2:**
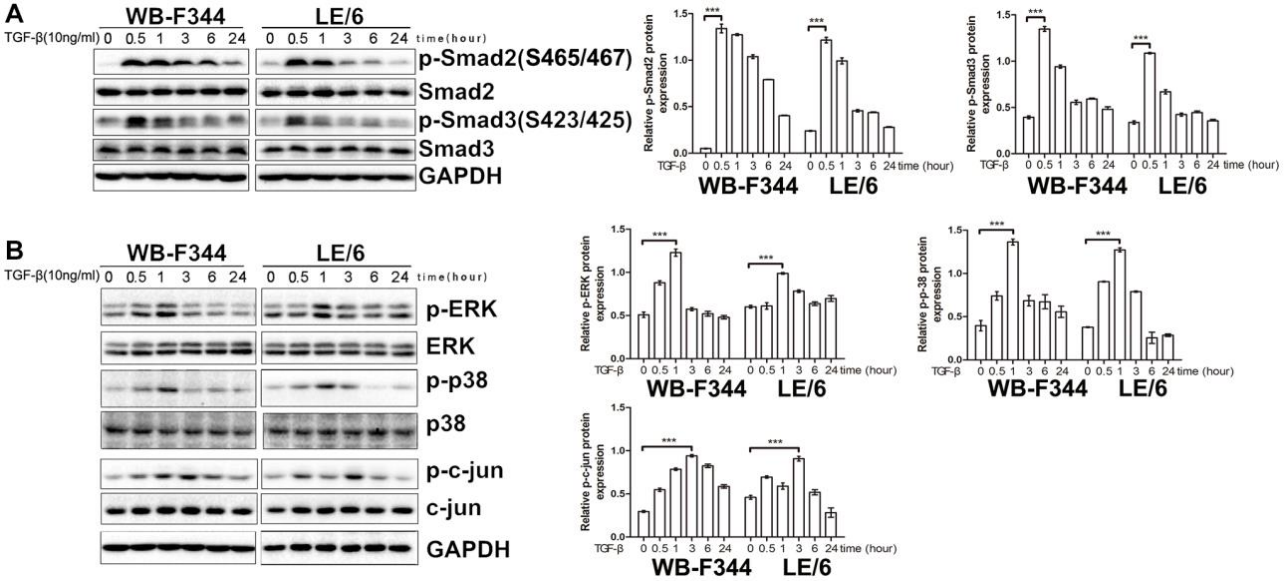
**Both Smad and MAPK signaling are activated by TGF-β in liver progenitor cells.** (**A**, **B**) LPCs (LE/6 and WB-F344 cells) were treated with TGF-β (10 ng/ml) for the indicated times, and the lysates were subjected to Western blot analyses with antibodies against the indicated proteins. Representative blot images of three independent experiments are shown, and GAPDH was used as a loading control.

We subsequently investigated the signaling cascades that contributed to the TGF-β-induced growth inhibition and EMT in LPCs. Previous studies have reported that Smad signaling is the classic downstream signaling cascade of TGF-β. We then carried out Western blot analyses, and the results showed that under stimulation with TGF-β, both Smad2 and Smad3 were phosphorylated in a time-dependent manner in WB-F344 and LE/6 cells, which suggested that canonical TGF-β downstream of Smad signaling was activated in LPCs (Figure 2A). In addition, Western blot analyses revealed that in both WB-F344 and LE/6 cells, Erk, JNK and p38 MAPK were phosphorylated in a time-dependent manner, which suggested that in LPCs, the major components of MAPK signaling (Erk, JNK and p38 MAPK signaling) are also activated by TGF-β. In detail, we found that the induced phosphorylation of Smad2 and 3 at the C-terminus peaked at 0.5 h to 1 h after TGF-β induction, whereas the TGF-β-induced phosphorylation of p-Erk, JNK and p38 MAPK peaked at 1–3 h after induction, accompanied by a reduction in phosphorylated Smad2 and 3 (Figure 2B).

### Suppression of TGF-β downstream of Erk, JNK or p38 MAPK signaling sensitizes LPCs to TGF-β-induced cytostatic effects

**Figure 3:**
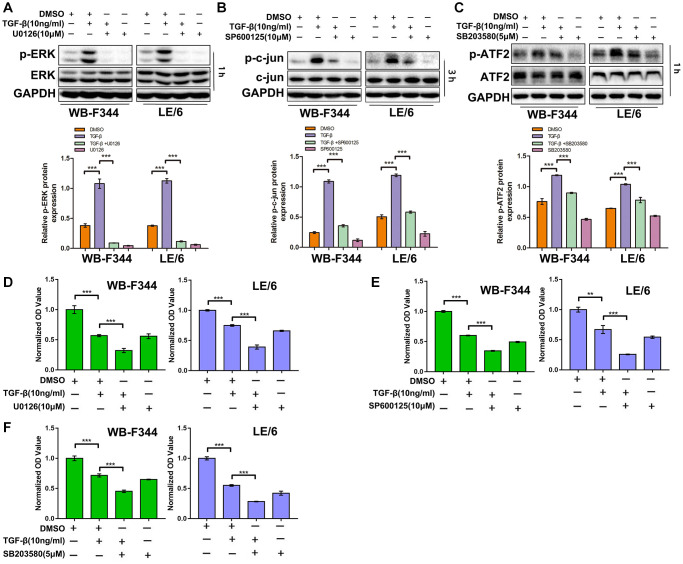
**Suppression of TGF-β downstream of Erk, JNK or p38 MAPK signaling strengthens the TGF-β-induced cytostatic effects in LPCs.** (**A**–**C**) WB-F344 and LE/6 cells were treated with TGF-β, U0126 (an Erk inhibitor), SP600125 (a JNK inhibitor), and/or SB203580 (a p38 MAPK inhibitor) as indicated, and Western blot analyses were carried out with antibodies against phospho-ERK and ERK (**A**), phospho-c-jun and c-jun (**B**), and phospho-ATF2 and ATF2 (**C**). GAPDH was used as a loading control. (**D**–**F**) WB-F344 and LE/6 cells were treated with TGF-β and/or the inhibitors as indicated for 3 days, after which CCK-8 analyses were performed. The normalized OD values of each group were compared, and the average OD values of three independent experiments are shown. One-way ANOVA was used for statistical analysis. The data are presented as the mean ± S.E.M. ^***^p < 0.001; ^****^p < 0.0001.

We found that TGF-β induced limited cytostatic effects in LPCs, and our previous studies demonstrated that Smad signaling contributed to these inhibitory effects on the growth of LPCs. To explore the role of TGFβ-MAPK signaling in LPCs, we treated LPCs with both TGF-β and MAPK inhibitors, including U0126 (an Erk inhibitor, 10 μM), SP600125 (a JNK inhibitor, 10 μM) and SB203580 (a p38 MAPK inhibitor, 5 μM), and then carried out Western blot and CCK-8 analyses. In LPCs, TGF-β activated Erk, JNK, and p38 MAPK signaling, which was indicated by the phosphorylation of Erk, c-jun and ATF-2, which was inhibited by U0126 (Figure 3A and Supplementary Figure 2), SP600125 (Figure 3B), and SB201580 (Figure 3C), respectively. The results of the CCK-8 assays revealed that in both WB-F344 and LE/6 cells, the inhibition of Erk signaling by U0126 augmented the suppressive effect of TGF-β on cell growth (Figure 3D). Similar effects were also found for the SP600125- or SB203580-treated LPCs (Figure 3E, 3F). Taken together, these results suggested that suppressing TGF-β downstream of MAPK signaling sensitizes LPCs to TGF-β-induced cytostatic effects.

### Inhibition of TGF-β-induced Erk, JNK or p38 MAPK signaling augmented TGF-β-mediated EMT in LPCs

**Figure 4:**
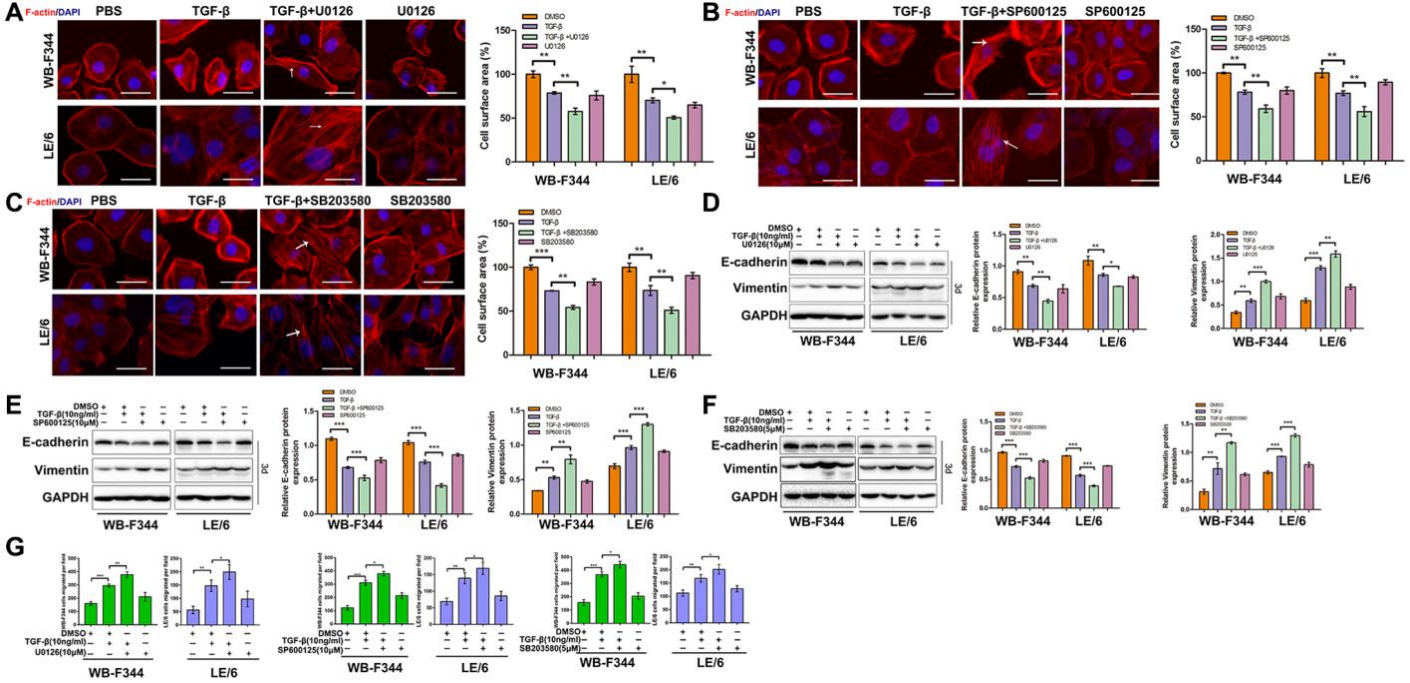
**Inhibition of TGF-β-induced Erk, JNK or p38 MAPK signaling augmented TGF-β-mediated EMT and motility in LPCs.** (**A**–**C**) Liver progenitor cell lines (WB-F344 and LE/6) were treated with TGF-β and/or kinase inhibitors as indicated for 3 days and then subjected to phalloidin staining for F-actin (red). DAPI (blue) was used to stain the cell nuclei. The white arrows indicate F-actin rearrangements. Scale bar, 25 μm. (**D**–**F**) WB-F344 and LE/6 cells were treated with TGF-β and/or kinase inhibitors as indicated for three days, and Western blot analyses were carried out with antibodies against E-cadherin and vimentin. Representative bands of three independent experiments are shown, and GAPDH was used as a loading control. (**G**) Cell motility analyses of LPCs treated with TGF-β and/or kinase inhibitors as indicated (for WB-F344 cells, TGF-β and/or kinase inhibitors were added for seven hrs, and for LE/6 cells, TGF-β and/or kinase inhibitors were added for 24 hrs). The average number of migrated cells per field in three independent experiments is shown. One-way ANOVA was used for statistical analysis. The data are presented as the mean ± S.E.M. ^*^p < 0.05; ^**^p < 0.01; and ^***^p < 0.001.

Our previous studies demonstrated that TGFβ induces EMT in LPCs through activating Smad signaling. To further investigate the role of TGF-β in activating Erk, JNK and p38 MAPK signaling in LPCs, we suppressed the TGF-β downstream effectors Erk, JNK and p38 MAPK via specific inhibitors in LPCs. The results showed that TGF-β induced obvious EMT after Erk, JNK or p38 MAPK signaling was inhibited in LPCs, which was shown by the elevated rearrangement of F-actin (Figure 4A–4C), decreased expression of the epithelial marker E-cadherin, increased expression of the mesenchymal marker vimentin (Figure 4D–4F and Supplementary Figure 3), and enhanced capacity for cell migration (Figure 4G and Supplementary Figure 4). These results suggested that TGF-β-induced Erk, JNK and p38 MAPK signaling plays an inhibitory role in the TGF-β-induced EMT of LPCs.

### TGF-β activated Erk, JNK and p38 MAPK signaling and inhibited TGFβ-Smad3 signaling in LPCs

**Figure 5:**
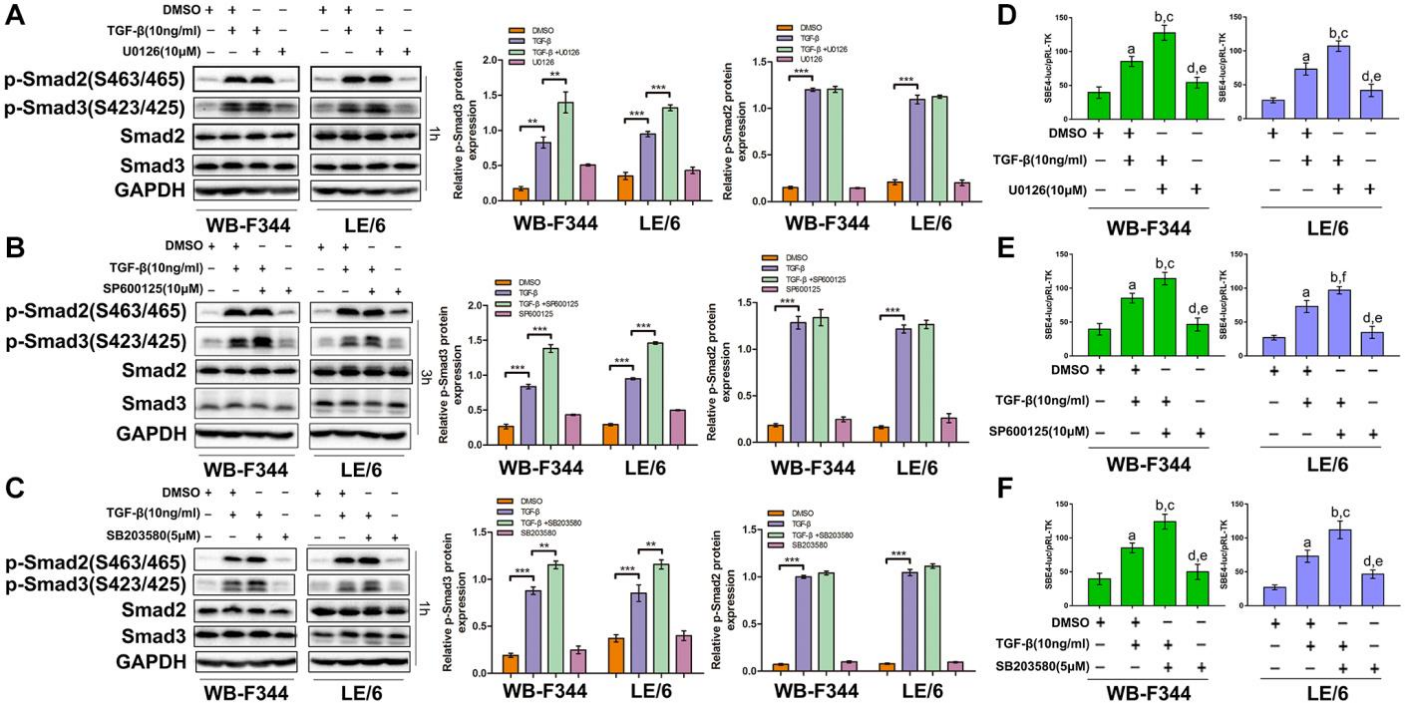
**TGF-β activated Erk, JNK and p38 MAPK signaling and inhibited TGFβ-Smad3 signaling in LPCs.** (**A**–**C**) LPCs were treated with TGF-β and/or kinase inhibitors as indicated, and the levels of phospho-Smad2 and Smad3 at the C-terminus and related total Smad2 and 3 proteins in the cells were analyzed via Western blotting. GAPDH was used as a loading control. (**D**–**F**) LPCs were cotransfected with pRL-TK and SBE4-luc and treated with TGF-β and/or kinase inhibitors as indicated. Luciferase activity was normalized to Renilla luciferase activity and is expressed as the means ± SEMs of triplicate measurements. The following comparisons of the bars were made: a, *P* < 0.01, second with first; b, *P* < 0.001, third with first; c, *P* < 0.01, third with second; d, *P* < 0.01, fourth with second; e, *P* < 0. 001, fourth with third; f, *P* < 0.05, third with second. The experiments were repeated three times. One-way ANOVA was used for statistical analysis. The data are presented as the mean ± S.E.M.

We found that TGF-β downstream of MAPK cascades suppressed TGFβ-induced cytostatic and EMT effects in LPCs. To explore whether TGFβ-MAPK signaling inhibits TGFβ-Smad signaling in LPCs, we treated LPCs with TGF-β and specific MAPK inhibitors, including U0126, SP600125 and SB203580, and carried out Western blotting to analyze the activation of Smad signaling in LPCs. Treatment with U0126, SP600125 or SB203580 had little effect on the phosphorylation of Smad2 in LPCs stimulated with TGF-β, whereas the TGF-β-induced phosphorylation of Smad3 in LPCs was elevated after treatment with MAPK inhibitors, including U0126 (Figure 5A), SP600125 (Figure 5B) or SB203580 (Figure 5C). These results suggested that in LPCs, TGFβ-MAPK signaling inhibited TGFβ-Smad3 signaling but did not alter the status of Smad2. To further confirm these findings, we performed luciferase reporter analyses, and the results showed that TGF-β activated SBE4-luc (a Smad3 binding element) in both WB-F344 and LE/6 cells, and this activation was further strengthened after the activation of Erk, JNK, or p38 MAPK was inhibited (Figure 5D–5F). Taken together, these results suggested that in LPCs, the signaling of the TGF-β downstream effectors Erk, JNK and p38 MAPK inhibited the activation of Smad signaling. In particular, TGF-β, which is downstream of Smad3 but not Smad2 signaling, was inhibited by TGFβ-MAPK signaling in LPCs.

### Inhibition of Smad signaling downstream of TGF-β abrogated TGF-β-induced cytostasis and EMT and strengthened MAPK signaling in LPCs

**Figure 6:**
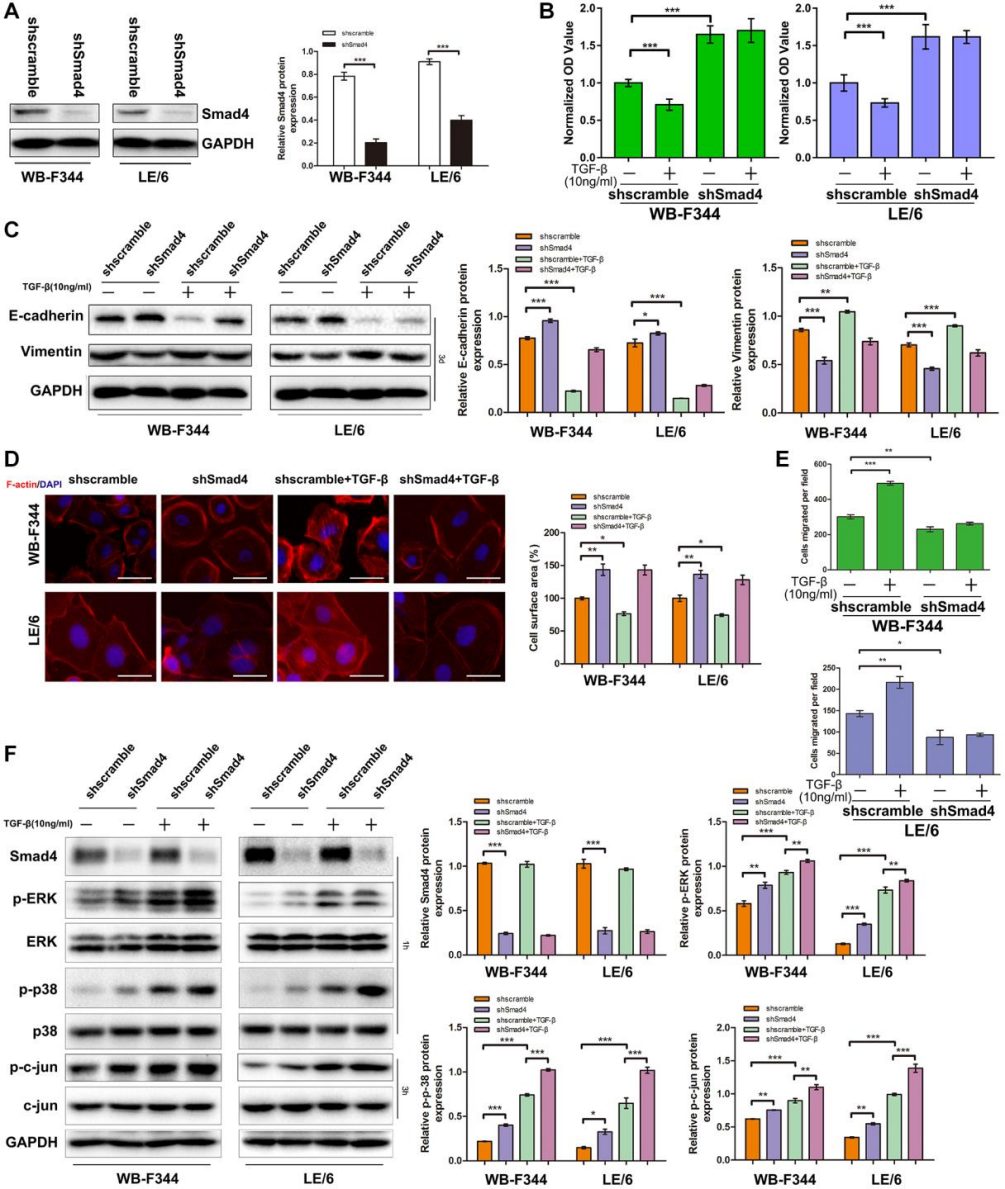
**Inhibition of Smad signaling downstream of TGF-β abrogated TGF-β-induced cytostasis and EMT and strengthened MAPK signaling in LPCs.** (**A**) Western blotting was used to measure the expression of Smad4 in WB-shSmad4, LE-shSmad4, and control cells (WB-shScramble or LE-shcramble cells). GAPDH was used as a loading control. (**B**) CCK-8 assays of WB-shSmad4, LE-shSmad4 and control cells before and after treatment with TGF-β (10 ng/ml, 3d). OD values were normalized to those of the control groups. (**C**) WB-shSmad4, LE-shSmad4, and control cells were treated with TGF-β (10 ng/ml, 3d) as indicated, and Western blot analyses were carried out with antibodies against E-cadherin and vimentin. GAPDH was used as a loading control. (**D**) WB-shSmad4, LE-shSmad4 and control cells were treated with TGF-β (10 ng/ml) for 3 days and then subjected to phalloidin staining for F-actin (red). DAPI (blue) was used to stain the cell nuclei. The white arrows indicate F-actin rearrangements. Scale bar, 25 μm. (**E**) Cell motility analyses of WB-shSmad4, LE-shSmad4 and control cells treated with TGF-β. The average number of migrated cells per field is shown. (**F**) Western blot analyses of Smad4, p-ERK, ERK, p-p38 MAPK, p38 MAPK, p-c-Jun and c-Jun expression in WB-shSmad4, LE-shSmad4, and control cells. GAPDH was used as a loading control. WB-shSmad4, LE-shSmad4 and control cells were treated with TGF-β (10 ng/ml) for 1 h or 3 h, respectively. These experiments were repeated three times. One-way ANOVA was used for statistical analysis. The data are presented as the mean ± S.E.M. ^*^p < 0.05; ^**^p < 0.01; and ^***^p < 0.001.

We then suppressed Smad signaling in LPCs through shRNA-mediated interference of Smad4 expression (Figure 6A and Supplementary Figure 5) and carried out CCK-8 analyses. The results showed that TGF-β-induced cytostasis was abrogated after Smad4 was knocked down in LPCs (Figure 6B). The results of Western blot analyses of EMT-related markers (Figure 6C), F-actin fluorescence staining (Figure 6D) and cell motility (Figure 6E and Supplementary Figure 6) in LPCs revealed that TGF-β-induced EMT and motility in LPCs were mitigated after Smad4 knockdown. In addition, the results of Western blot analyses of LPCs showed that knockdown of Smad4 strengthened TGF-β-induced MAPK signaling, as indicated by the phosphorylation of Erk, c-jun and p38 MAPK (Figure 6F). These results suggested that TGF-β-induced Smad signaling suppressed downstream MAPK signaling in LPCs.

### TGF-β downstream MAPK signaling promotes linker phosphorylation of Smad3 in liver progenitor cells

**Figure 7:**
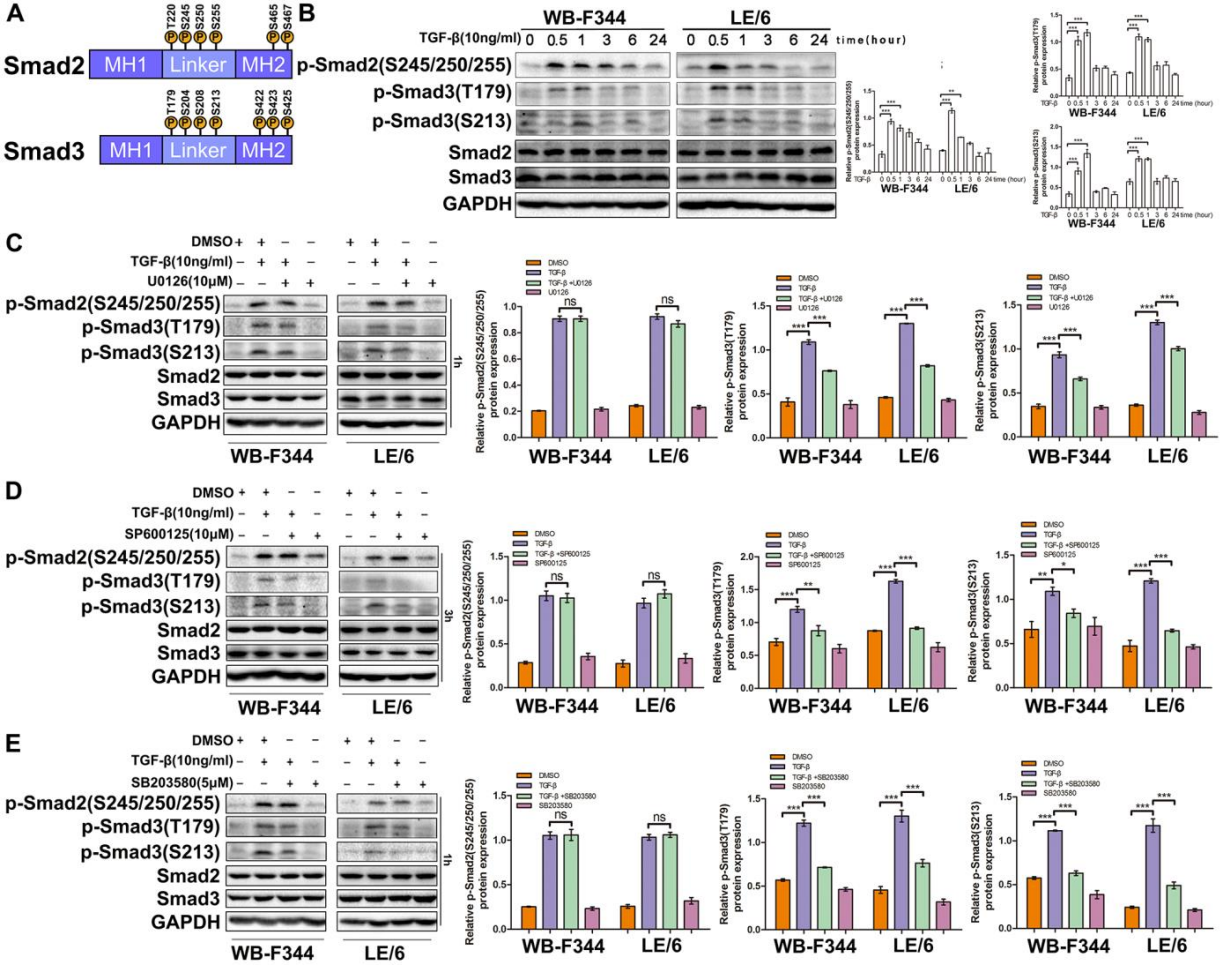
**TGF-β downstream of MAPK signaling promotes linker phosphorylation of Smad3 in liver progenitor cells.** (**A**) Schematic representation of the phosphorylation sites in Smad2 and Smad3. (**B**) WB-F344 and LE/6 cells were treated with TGF-β (10 ng/ml) for the indicated times, and lysates were subjected to Western blot analyses with antibodies against the indicated phospho- and total Smad proteins. GAPDH was used as a loading control. (**C**–**E**) WB-F344 and LE/6 cells were treated with TGF-β (10 ng/ml, 1 h) and/or kinase inhibitors as indicated for 1 h or 3 h, and Western blot analyses were carried out with antibodies against the indicated phospho- and total Smad proteins. GAPDH was used as a loading control. The experiments were repeated three times, and representative images are shown.

Previous studies have reported that in mammalian cells, both Smad2 and Smad3 can be phosphorylated at their linker and C-terminal regions through different upstream signals, and Smad isoform signals play distinct roles in the proliferation, EMT, motility, and carcinogenesis of cells [15] (Figure 7A and Supplementary Figure 7). To explore the occurrence of Smad phosphoisoform signaling in LPCs, we treated LPCs with TGF-β and carried out Western blot analysis. The results showed that TGF-β induced linker phosphorylation of Smad2 and Smad3 in LPCs (Figure 7B). In addition, blocking the activity of Erk, JNK or p38 MAPK with specific inhibitors (U0126, SP600125, and SB203580, respectively) reduced the TGF-β-induced linker phosphorylation of Smad3, whereas the TGF-β-induced linker phosphorylation of Smad2 was not influenced by the inhibition of Erk, JNK or p38 MAPK signaling in LPCs (Figure 7C–7E). Taken together, these results suggested that TGF-β-MAPK signaling contributed to the linker phosphorylation of Smad3 in LPCs.

### Smad3 phosphorylation affects TGF-β-induced cytostasis and EMT in LPCs

**Figure 8:**
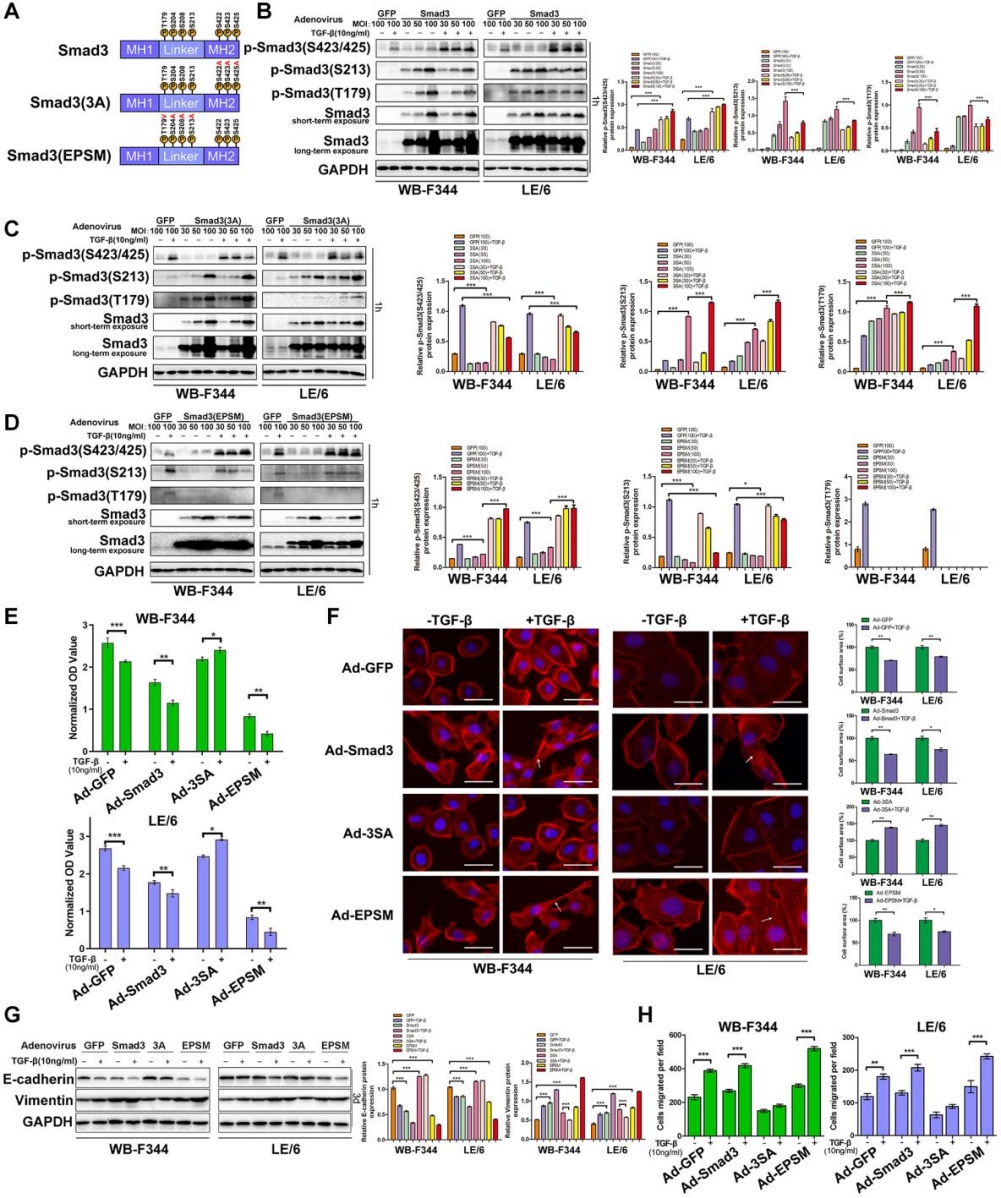
**Smad3 phosphorylation affects TGF-β-induced cytostasis and EMT in LPCs.** (**A**) Schematic representation of the phosphorylation sites in Smad3, the corresponding C-terminal region (3SA) and the corresponding linker region (EPSM). (**B**–**D**) WB-F344 and LE/6 cells were infected with adenoviruses carrying GFP (Ad-GFP), Smad3 (Ad-Smad3), or Smad3 (Ad-3SA or Ad-EPSM) at the indicated MOIs and were treated with TGF-β (10 ng/ml) for 3 1 h. Cell lysates were subjected to Western blot analyses with antibodies against the indicated phospho- and total Smad3 proteins. GAPDH was used as a loading control. (**E**) WB-F344 (upper panel) and LE/6 (lower panel) cells were infected with adenoviruses carrying Smad3 or its mutants, treated with TGF-β (10 ng/ml, 3d), and subjected to CCK-8 analyses. OD values were measured and compared between groups as indicated. (**F**) WB-F344 and LE/6 cells infected with adenoviruses carrying Smad3 or its mutants as indicated were treated with TGF-β (10 ng/ml, 3d) and then subjected to phalloidin staining for F-actin (red). DAPI (blue) was used to stain the cell nuclei. The white arrows indicate F-actin rearrangements. Scale bar, 25 μm. (**G**) WB-F344 and LE/6 cells infected with adenoviruses carrying Smad3 or its mutants as indicated were treated with TGF-β (10 ng/ml) for 3 days, and Western blot analyses were performed with antibodies against E-cadherin and vimentin. GAPDH was used as a loading control. (**H**) Cell motility analyses of WB-F344 and LE/6 cells infected with adenovirus carrying Smad3 or its mutants as indicated and control cells treated with TGF-β. The average number of migrated cells per field is shown. The experiments were repeated three times. Two-tailed Student’s *t*-tests were used for statistical analysis. The data are presented as the mean ± S.E.M. ^*^p < 0.05; ^**^p < 0.01; and ^***^p < 0.001.

**Figure 9:**
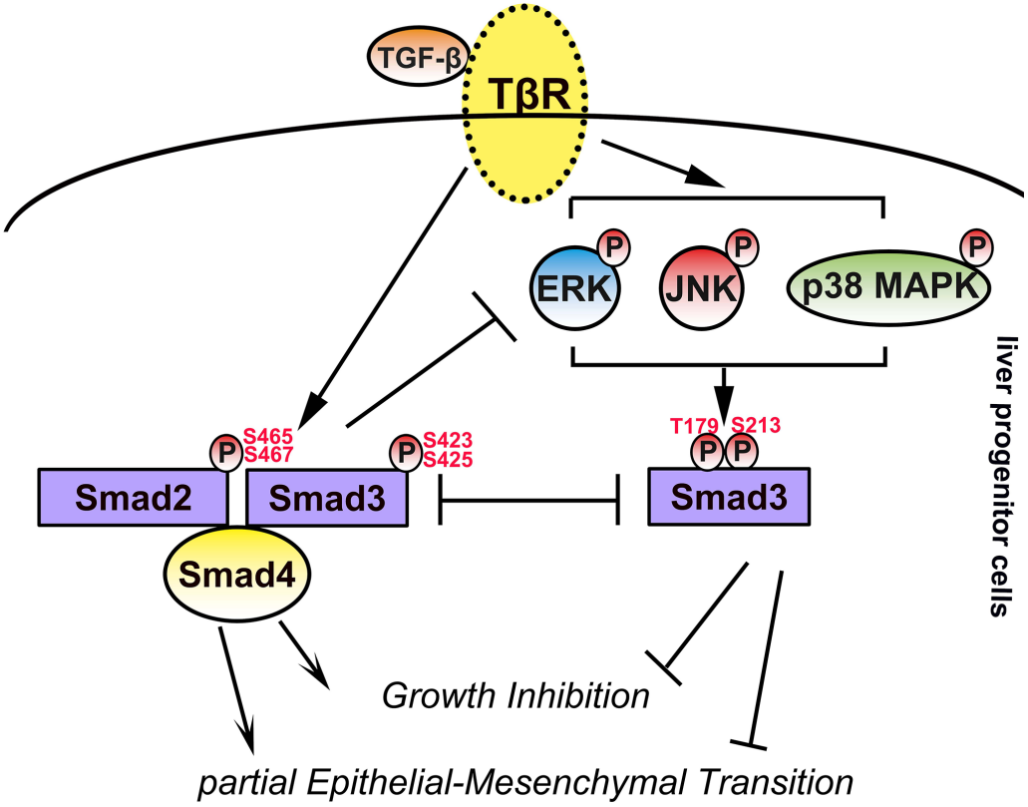
**Schematic illustration of this study.** In LPCs, TGF-β activated Smad and MAPK signaling, and activated MAPK signaling contributed to linker phosphorylation of Smad3 at T179 and S213. TGF-β-activated Smad signaling contributes to the cytostasis and EMT of LPCs, whereas TGF-β-activated MAPK signaling and phosphorylation of Smad3 at T179 and S213 have opposite effects.

To explore the roles of the linker and C-terminal phosphorylation of Smad3 in LPCs, we used recombinant adenoviruses harboring constitutively expressed wild-type Smad3 (Ad-Smad3) or its mutant at the C-terminal (Ad-3SA) or linker region (Ad-EPSM), in which all serine/threonine phosphorylation sites were replaced with alanine (A) or valine (V, Figure 8A and Supplementary Figures 8 and 9). An adenovirus carrying green fluorescent protein (Ad-GFP) was used as a control in the present study. We first infected Ad-Smad3, Ad-S3A, Ad-EPSM, and Ad-GFP into WB-F344 and LE-6 cells, and the Western blotting results showed that after infection of LPCs with Ad-Smad3, the phosphorylation of Smad3 at both the C-tail and linker region was elevated in a multiplicity of infection (MOI)-dependent manner (Figure 8B). In addition, infection of LPCs with Ad-3SA or Ad-EPSM caused a reduction in the C-terminus (Figure 8C) and linker phosphorylation of Smad3 (Figure 8D), respectively, in the presence or absence of TGF-β. Moreover, infection of LPCs with Ad-3SA promoted TGF-β-induced linker phosphorylation of Smad3 (Figure 8C), and infection of LPCs with Ad-EPSMs increased the C-terminal phosphorylation of Smad3, as indicated by stimulation with TGF-β (Figure 8D). We then carried out cell proliferation analyses, and the results showed that infection with Ad-Smad3 in LPCs augmented TGF-β-induced cytostasis, and infection with Ad-EPSMs markedly enhanced the cytostatic effect of TGF-β. In contrast, LPCs infected with Ad-3SA were resistant to TGF-β-induced cytostasis, and TGF-β even promoted the proliferation of these cells (Figure 8E). The results of EMT-related analyses revealed that infection of LPCs with Ad-3SA abrogated EMT and cell migration in the presence and absence of TGF-β, whereas infection with Ad-EPSMs enhanced the effects of TGF-β-induced EMT in LPCs (Figure 8F–8H and Supplementary Figure 10). Collectively, these results suggested that overexpression of mutated Smad3 at linker phosphorylation sites intensifies TGF-β-induced cytostasis and EMT, mimicking the effects of MAPK inhibition in LPCs, whereas mutation of Smad3 at the C-terminus caused LPCs to blunt TGF-β-induced cytostasis and EMT, similar to the effects of Smad4 knockdown (Figure 9).

## DISCUSSION

The activation and expansion of LPCs, maintenance of progenitor features, and effects of LPCs in liver regeneration, fibrosis and carcinogenesis are regulated by cytokines in the presence of LPCs [2]. TGF-β is a pivotal cytokine in the microenvironment of injured and fibrotic livers. However, one of the classic roles of TGF-β in mammalian cells is to induce cytostasis, and TGF-β-induced cytostasis was also demonstrated by our group and others in LPCs [16]. However, how LPCs survive and expand in fibrotic conditions induced by TGF-β has still been explored. In the present study, we confirmed that TGF-β induced growth inhibition in LPCs, whereas this effect was limited. These results are consistent with the findings of a previous study in which LPCs were not sensitive to TGF-β-induced cytostasis, and it was also reported that the intrinsic high expression of Smad6, an inhibitory Smad, contributed to the insensitivity of LPCs to TGF-β [16]. In addition, previous studies have shown that extrinsic cytokines such as HGF and EGF, both of which can activate mitogenic signaling in cells, can counterbalance the effect of TGF-β on LPCs [17, 18]. In this study, we confirmed that mitogenic MAPK signaling pathways, such as the Erk, JNK and p38 MAPK signaling pathways, were activated by TGF-β in LPCs and that TGF-β-activated MAPK signaling and Smad signaling antagonistically regulated the proliferation of LPCs. Considering that, in LPCs, the TGF-β-induced phosphorylation of p-Erk, JNK and p38 MAPK peaks after the phosphorylation of Smad2 and Smad3 at the C-terminus, TGF-β-induced MAPK signaling might be the consequence of TGF-β-induced Smad signaling and functional suppression of TGFβ-Smad signaling. In addition, these results suggest that mitogenic TGFβ-MAPK signaling may help LPCs overcome the cytostatic effect caused by TGFβ-Smad signaling and promote the survival and expansion of LPCs in the fibrotic environment.

The EMT contributes to cell growth, stemness, fibrosis, and cancer progression in different contexts [19, 20]. In this study, we discovered that TGF-β downstream of Smad and MAPK signaling antagonizes the EMT of LPCs and that the balance between Smad and MAPK signaling leads to the insensitivity of LPCs to TGF-β-induced EMT. This finding may be cellular specific because TGF-β-induced MAPK signaling was also found in hepatocytes, and previous studies reported that MAPK signaling was coordinated with Smad signaling in inducing EMT in hepatocytes and made hepatocytes sensitive to TGF-β-induced EMT [21]. On the basis of our previous study, we reported that LPCs undergo partial EMT, and autocrine TGF-β in LPCs contributes to these states [12]. In addition, another previous study demonstrated that LPCs stably maintain their progenitor phenotypes [22]. Moreover, a recent study revealed that TGF-β-induced partial EMT in LPCs is a step in hepatocyte differentiation, and c-Met signaling restrains these effects [17]. Taken together, our findings in this study and the results of these reports suggested that the balance of TGF-β downstream of Smad and MAPK signaling contributes to the maintenance of partial EMT states and stable progenitor phenotypes in LPCs, where TGF-β signaling is autocrine activated.

In this study, we discovered that in LPCs, TGF-β induced the expression of phosphoisoforms of Smad2 at both the C-terminus (pSmad2C) and linker region (pSmad2L) and phosphorylated Smad3, including S423 and 425 at the C-terminus as well as at T179 and S213 in the linker region; moreover, we did not find that TGF-β induced the phosphorylation of Smad3 at S204 and 208 in LPCs (data not shown). To our knowledge, our study is the first to delineate the roles and consequences of TGF-β-induced Smad phosphoisoforms in LPCs, and the pattern of TGF-β-induced phosphorylation of Smad2 and Smad3 may be context specific. Previous studies have reported that in HaCaT (an epidermal cell line) and Mv1Lu (a lung epithelial cell line) cells, TGF-β induces the phosphorylation of Smad3 at the C-terminus and linker region of Thr179, S204 and 208, whereas it does not phosphorylate Smad3 at S213 [23]. Previous studies reported that the TGF-β-induced phosphorylation of Smad3 at S204 and S208 was dependent on the activity of GSK3β and CDKs, whereas the activation of Erk was not associated with this phosphorylation. These mechanisms may account for the characteristic TGF-β-induced phosphorylation of Smad3 in LPCs.

In this study, we demonstrated that TGF-β-activated MAPK signaling contributed to the phosphorylation of Smad3 at T179 and S213 but did not affect linker phosphorylation of Smad2. In addition, we proved that TGF-β downstream of Smad and MAPK signaling antagonizes Smad3 via its C-terminal and linker regions, whereas this antagonistic effect has no effect on the phosphoisoform of Smad2 in LPCs. Previous studies reported that JNK is an inducer of Smad3 phosphorylation at S213 (pSmad3L (S213)) in hepatocytes [24], which is consistent with our findings in LPCs. In addition to JNK, TGF-β-activated Erk and p38 MAPK also contributed to the phosphorylation of Smad3 at S213 and T179.

In the liver, phosphorylated Smad2 and 3 play different roles at different sites: pSmad2L and pSmad2C activate fibrotic signaling, and pSmad3C activates cytostatic signaling, whereas pSmad3L (S213) plays mitogenic and oncogenic roles. In addition, pSmad3L (T179) has both mitogenic and migratory functions. However, these conclusions are based on findings in hepatocytes and hepatic stellate cells, and the roles and regulation of Smad phosphoisoforms in LPCs are still being explored. In this study, mutation analyses of Smad3 phosphorylation sites revealed that linker phosphorylation of Smad3 suppressed TGF-β-induced cytostasis, EMT and migration in LPCs, whereas Smad3 phosphorylated at the C-terminus exhibited cytostatic effects, promoting EMT and migration. These results were consistent with previous findings in hepatocytes. In addition, sustained pSmad3L (S213) in hepatocytes has been demonstrated to be carcinogenic and is induced by oncogenic signaling, such as HBV antigen X (HBx)-activated JNK [25]. In the present study, we found that TGF-β induces pSmad3L (S213) in LPC, and we and others have demonstrated that TGF-β can induce malignant transformation of LPCs independently or enhance oncogenic HBx-JNK signaling [9, 13]. These results suggested that TGF-β-induced sustained pSmad3L (S213) may contribute to the malignant transformation of LPCs. In addition, previous studies have reported that pSmad3L (T179) signaling plays important roles in stem cell maintenance [26]. However, whether these mutations contribute to the maintenance of progenitor phenotypes in LPCs needs to be explored in the future.

## CONCLUSION

In conclusion, we reported that Smad3 and MAPK signaling downstream of TGF-β play antagonistic roles in the growth, EMT and motility of LPCs. This antagonistic effect contributed to the TGF-β-induced phosphorylation of Smad3 in LPCs and may account for how LPCs overcome the cytostatic effect of TGF-β and maintain partial EMT against the background of TGF-β signaling. In addition, this antagonistic mechanism may be important for the survival and maintenance of progenitor phenotypes of LPCs in the fibrotic microenvironment.

## MATERIALS AND METHODS

### Cell lines, cell culture, and reagents

The liver progenitor cell lines LE/6 and WB-F344 were used in this study. The origin, characteristic phenotypes and cell culture of these two cell lines were described in our previous study [12]. The generation and culture of LPCs with shRNA-mediated knockdown of Smad4 (WB-shSmad4 and LE-shSmad4 cells) as well as their corresponding control cells (WB-shSc and LE-shSc cells) were performed as described previously [10]. The cytokines and kinase inhibitors used in this study are listed in Supplementary Table 1. The antibodies used in this study are listed in Supplementary Table 2.

### Recombinant adenovirus production and transfection

The recombinant adenoviral Smad3 (Ad-Smad3), its C-terminal mutant (Ad-3SA) or its linker region (Ad-EPSM), in which all serine/threonine phosphorylation sites were replaced with alanine (A) or valine (V) fragments, was subcloned and inserted into the vector pHBAd-MCMV-GFP (Supplementary Figure 4, HanBio, Shanghai, China). Recombinant adenoviruses were amplified and purified by CsCl_2_ ultracentrifugation and quantified by TCID50. Ad-GFP was used as a control virus. The cells were infected at a multiplicity of infection (MOI) of 30–100 viral particles per cell as indicated. The infection efficiency of the adenovirus was monitored by evaluating green fluorescence, and the expression levels of Ad-Smad3, Ad-3SA and Ad-EPSM were confirmed by Western blot analyses.

### Cell viability assays

The viability of the LPCs was analyzed by CCK-8 assays [27]. Briefly, LPCs were seeded in 96-well plates at 800 cells/100 μl of medium per well. The attached cells were serum starved overnight (for more than 12 h) and incubated with reagents (DMSO, TGF-β, U0126, SP600125, or SB203580) as indicated for 3 days, after which cell viability was measured via a colorimetric assay using a CCK-8.

### Luciferase reporter analyses

Luciferase reporter analyses of the SBE4-luc reporter were performed as described previously [10, 12]. Briefly, 5 × 10^4^ cells per well were seeded, and the attached cells were cotransfected with SBE4-luc (0.48 μg) or pRL-TK (0.02 μg) plasmids through jetOPTIMUS (Polyplus-transfection, Illkirch-Graffenstaden, France). Six hours after transfection, the cells were cultured in fresh medium supplemented with 1% fetal bovine serum and incubated with the reagents as indicated. Then, the luciferase activity of the cells was detected via the Dual-Luciferase Reporter Assay System (Promega, Madison, WI, USA).

### Phalloidin staining of F-actin in cells

Phalloidin staining for the detection of F-actin in LPCs was performed as described previously [12]. Briefly, cells were cultured on coverslips in 6-well culture dishes and subjected to serum starvation overnight. The cells were then incubated with chemicals dissolved in culture medium as indicated, fixed in 4% paraformaldehyde, washed with 0.1% Triton X-100, and incubated with Alexa Fluor 555-conjugated phalloidin (Actin-Tracker Red-555, Beyotime Institute of Biotechnology, Shanghai, China). The nuclei were stained with 4’,6-diamidino-2-phenylindole (DAPI; Wuhan Servicebio Technology Co., Ltd., Wuhan, China), and fluorescence staining was performed on the cells with a Nikon Digital Eclipse C1 system (Nikon Corporation, Tokyo, Japan).

### Cell motility assay

Cell motility analyses were carried out in transwells as described previously [12]. The motility of the cells was detected in 24-well plates. Briefly, 1 × 10^4^ cells in 250 μL of cell culture medium supplemented with 0.2% FBS were seeded into the upper chamber, and 500 μL of medium supplemented with 10% FBS was added to the lower well of a 24-well transwell plate (8 μm pore size, Corning, NY, USA). Chemicals were added to both the upper and lower chambers. After 7 hrs (WB-F344 cells) or 24 hrs (LE/6 cells), the cells in the upper chamber were scraped off the cells on the top side of the chamber, and the migrated cells in the chamber were fixed in 4% paraformaldehyde, stained with 1% crystal violet, and counted with Image-Pro Plus (Media Cybernetics, Inc., Bethesda, MD, USA).

### Western blot analyses

Western blot analyses were carried out as described previously [4, 28]. Briefly, LPCs were lysed in radioimmunoprecipitation assay (RIPA) lysis buffer (P0013D, Beyotime) supplemented with protease inhibitors (Roche, Basel, Switzerland) and phosphatase inhibitors (PhoSSTOP Cocktail Tablets, Roche) on ice, after which the concentration of the proteins was measured via BCA protein assay kits (Beyotime). Equal amounts of cell lysate were then mixed with sodium dodecyl sulfate (SDS) loading buffer, denatured, loaded and separated via SDS–polyacrylamide (SDS–PAGE) gel (Bio-Rad, Bio-Rad, Hercules, CA, USA). After separation, the proteins were transferred to PVDF membranes (Roche), after which the proteins were incubated with primary antibodies overnight at 4°C. After incubation, the membrane was washed with TBST 3 times (5 min each) and then incubated for 1 h with a horseradish peroxidase (HRP)-labeled secondary antibody at 37°C. Finally, the membrane was washed again in TBST 3 times, reactive bands on the membrane were detected by enhanced chemiluminescence (ECL) reagent (Pierce Protein Biology, Thermo Fisher Scientific, Waltham, MA, USA), and the signals were visualized by an image labeling imaging system (Bio-Rad).

### Statistical analysis

The data are reported as the mean ± SEM of independent experiments. Student’s *t*-test was used for statistical analyses for comparisons of cell viability and motility between groups, luciferase reporter analyses and Western blotting assays, and the statistical analysis was performed with Prism 8.0 (GraphPad Software, La Jolla, CA, USA). *P* < 0.05 was considered to indicate statistical significance.

### Availability of data and materials

The data that support the findings of this study are available from the corresponding author (ZD) upon reasonable request.

## Supplementary Materials

Supplementary Figures

Supplementary Tables
