# Supplementary material for: TGF-β downstream of Smad3 and MAPK signaling antagonistically regulate the viability and partial epithelial–mesenchymal transition of liver progenitor cells
Source: Aging (Albany NY). 2024 Apr 5;16(7):6588–612. doi: 10.18632/aging.205725 (PMC11042936; doi:10.18632/aging.205725)
Supplement: Supplementary Figures [file aging-16-205725-s001.pdf]

SUPPLEMENTARY FIGURES

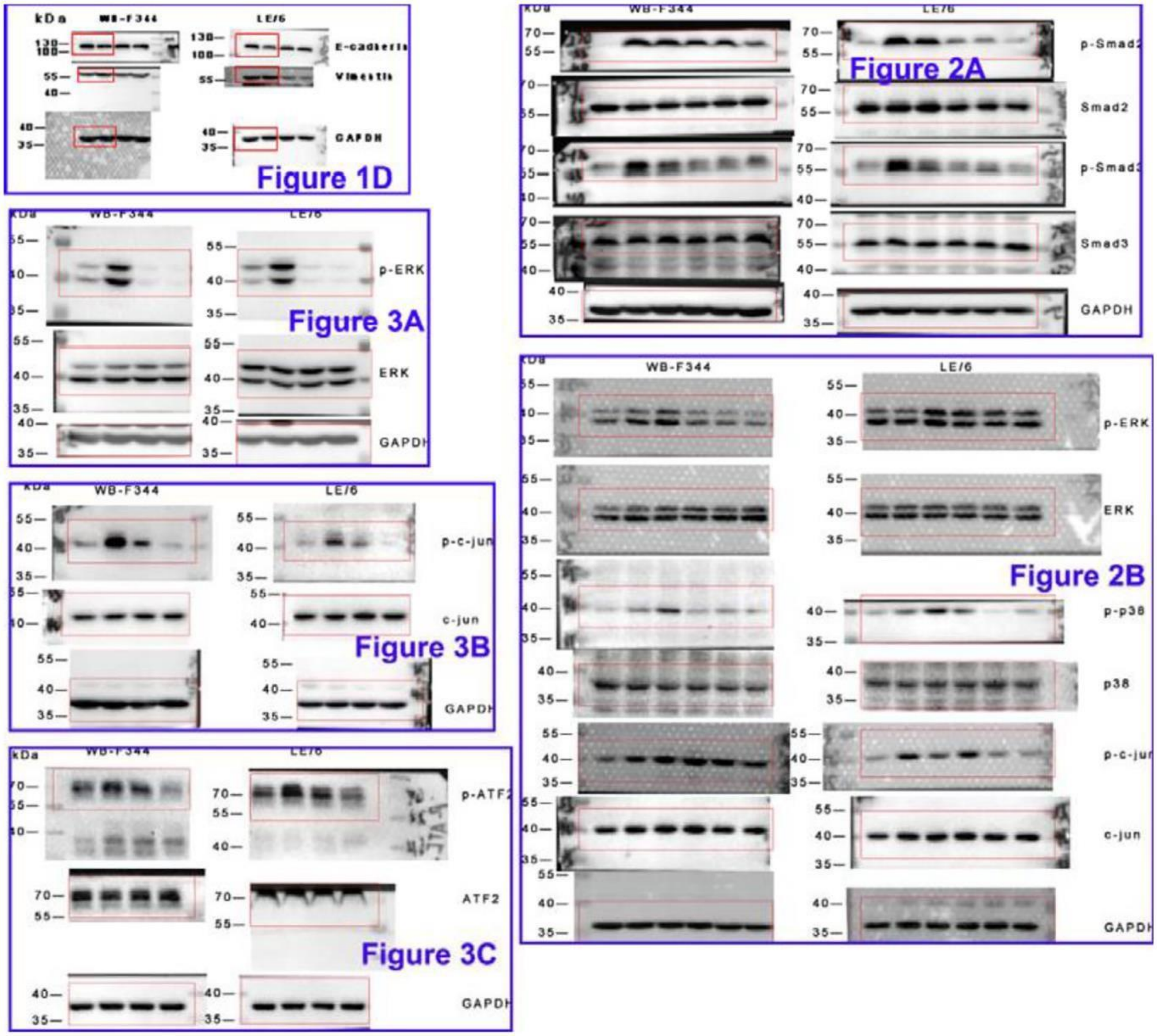

Supplementary Figure 1. Unprocessed Western blots, related to Figures 1–3. Western blot scanned films. Red boxes indicate lanes shown in the figures.

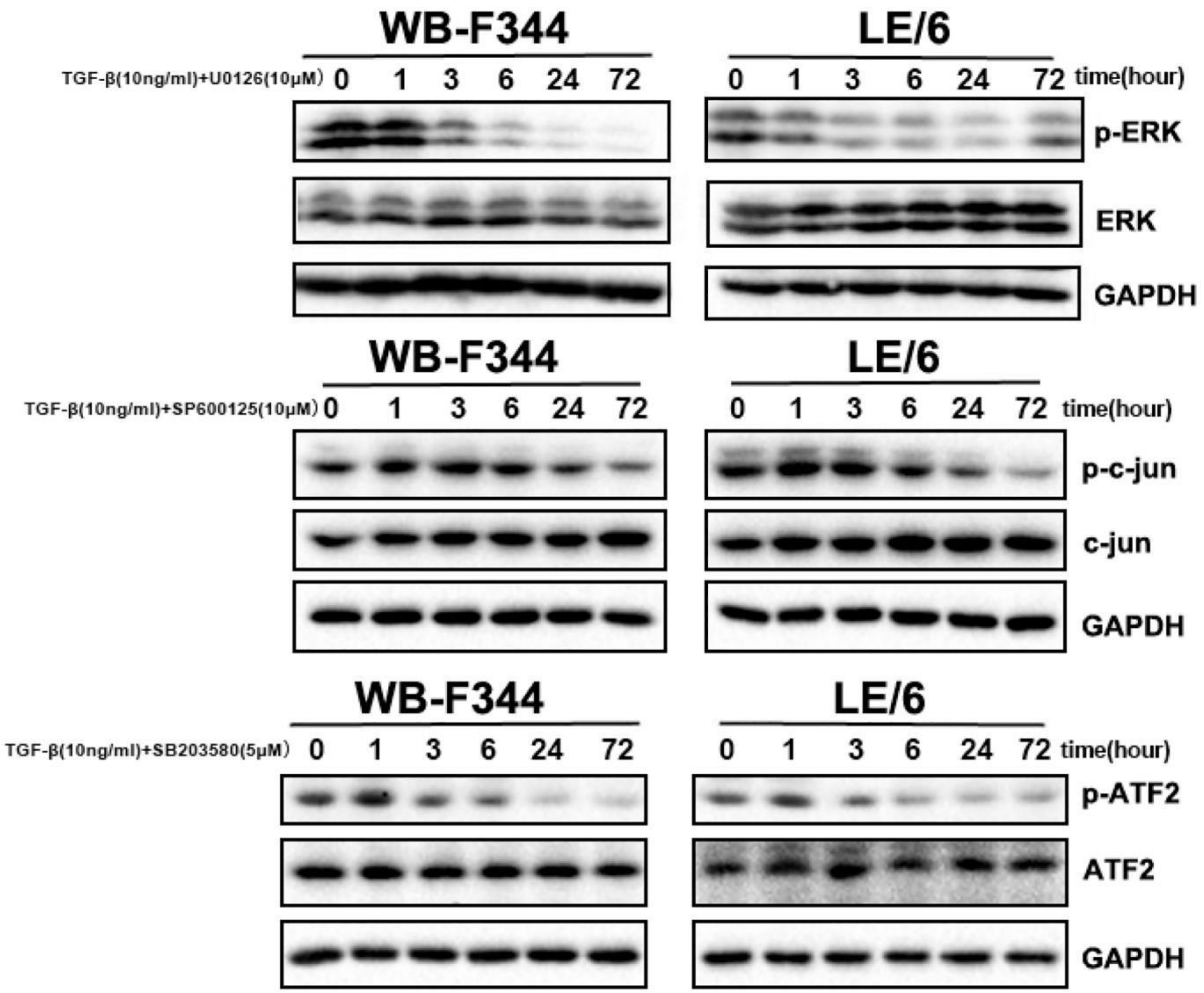

Supplementary Figure 2. LE/6 and WB-F344 cells were treated with TGF-β (10 ng/ml) plus MAPK inhibitors as indicated for up to 72 hrs, and the lysates were subjected to Western blot analyses with antibodies against the indicated proteins. Representative blot images of three independent experiments are shown, and GAPDH was used as a loading control.

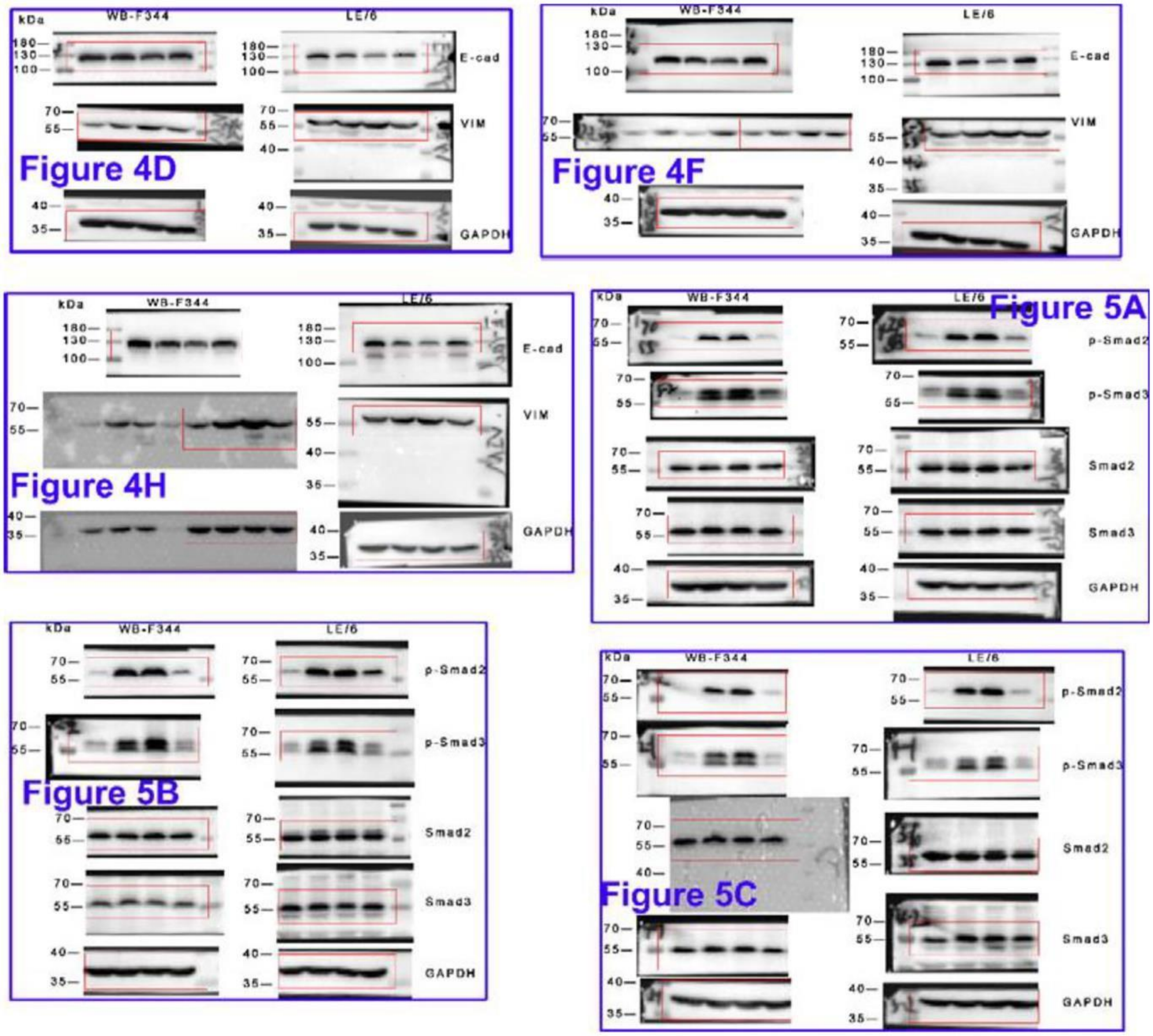

Supplementary Figure 3. Unprocessed Western blots, related to Figures 4 and 5. Western blot scanned films. Red boxes indicate lanes shown in the figures.

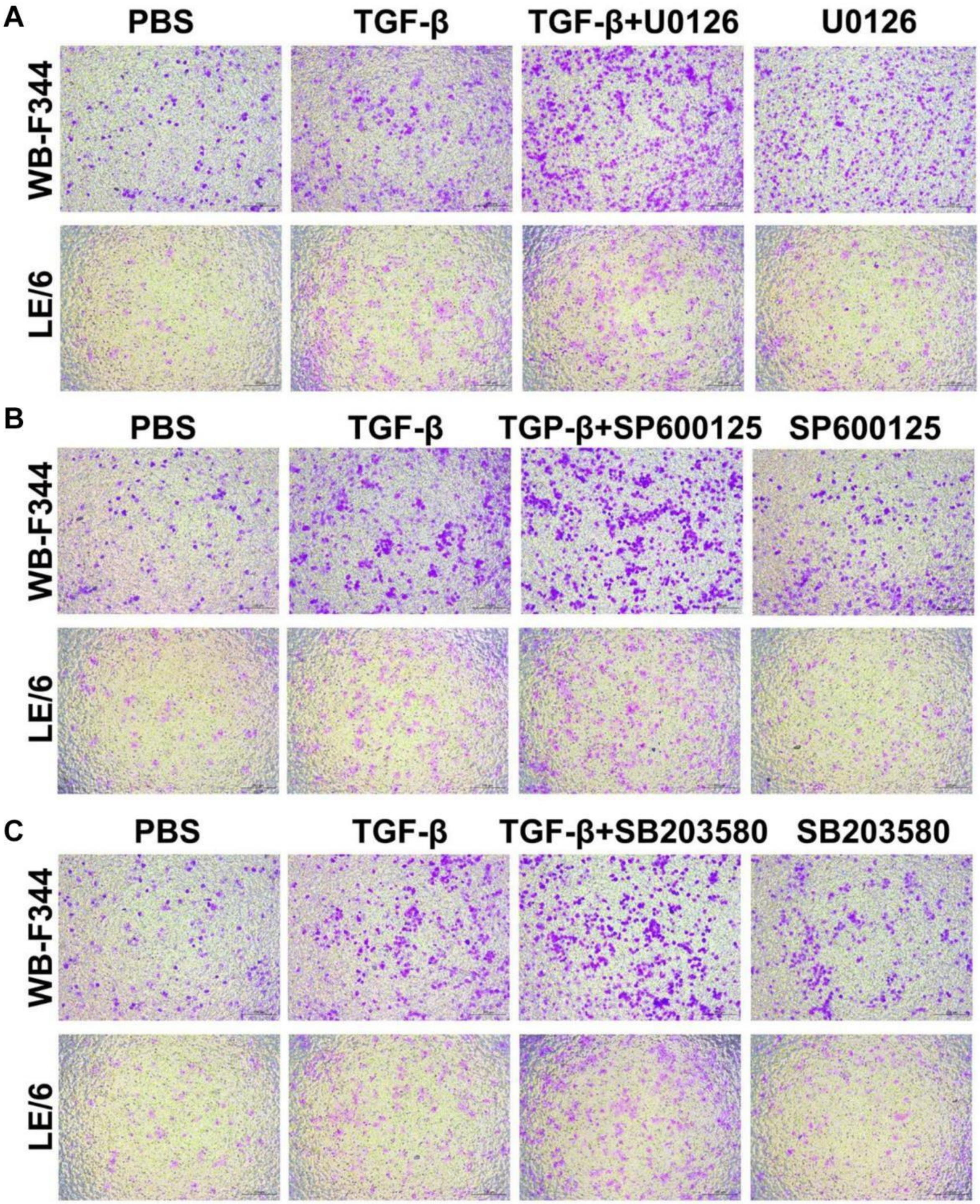

Supplementary Figure 4. Inhibition of Erk, JNK or p38 MAPK signaling augmented TGF-β mediated migration in LPCs, related to Figure 4. Representative images of transwell cell motility assays as described in Figure 4E (**A**); Figure 4G (**B**); and Figure 4I (**C**).

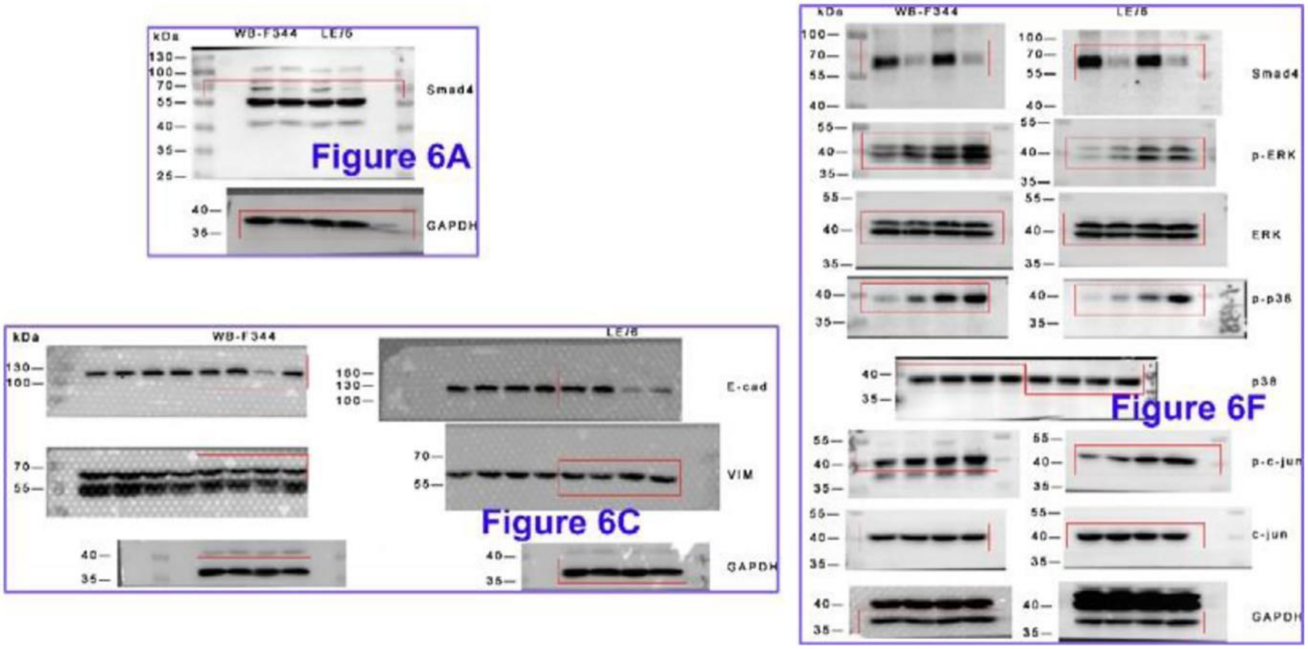

Supplementary Figure 5. Unprocessed Western blots, related to Figure 6. Western blot scanned films. Red boxes indicate lanes shown in the figures.

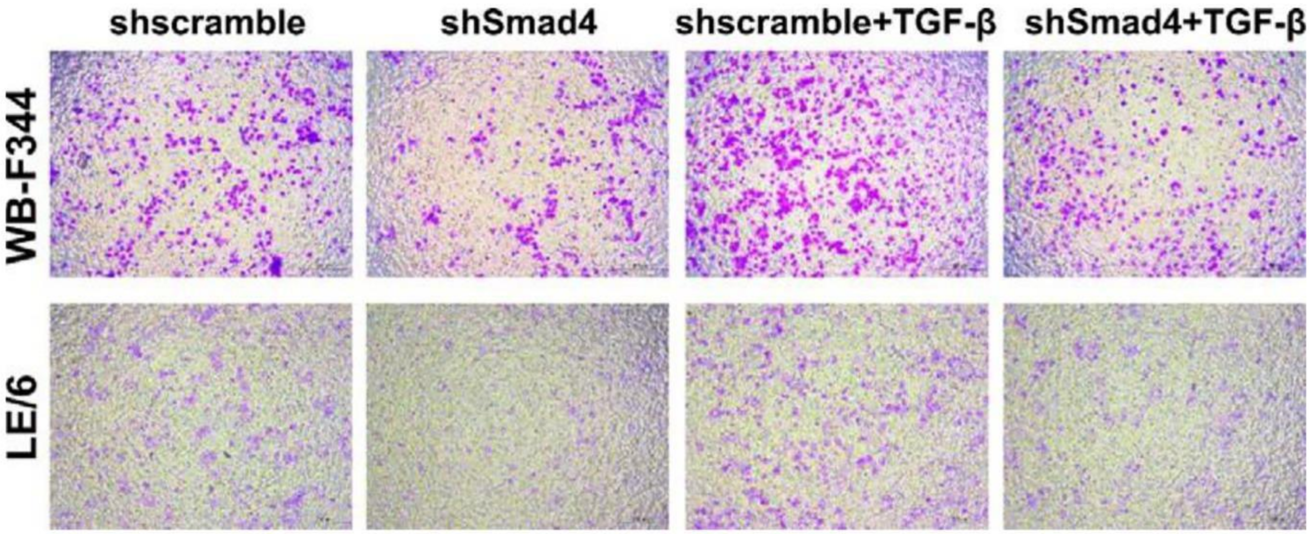

Supplementary Figure 6. Inhibition of TGF-β downstream Smad signaling abrogated TGF-β induced migration of LPCs, related to Figure 6. Representative images of cell motility assays as described in Figure 6E.

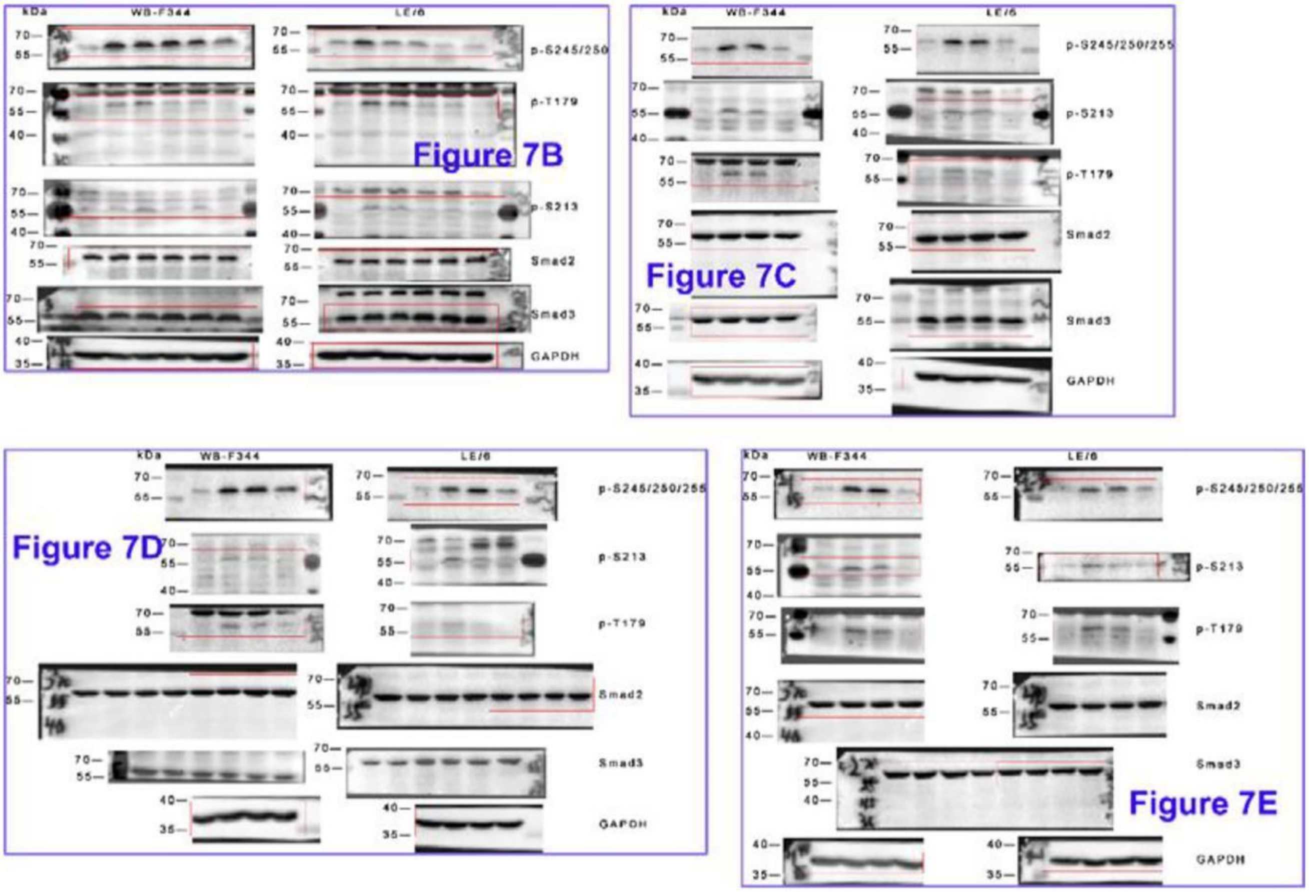

Supplementary Figure 7. Unprocessed Western blots, related to Figure 7. Western blot scanned films. Red boxes indicate lanes shown in the figures.

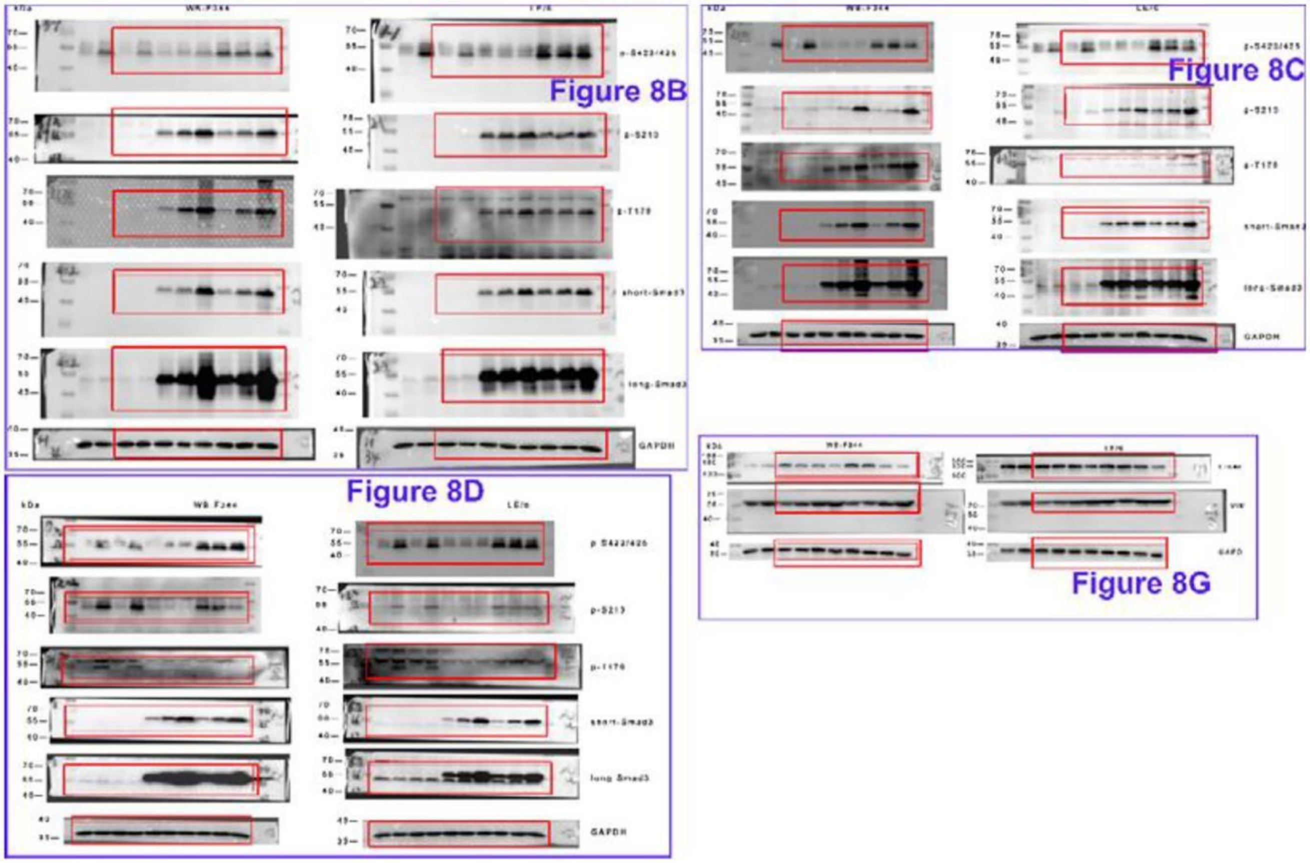

Supplementary Figure 8. Unprocessed Western blots, related to Figure 8. Western blot scanned films. Red boxes indicate lanes shown in the figures.

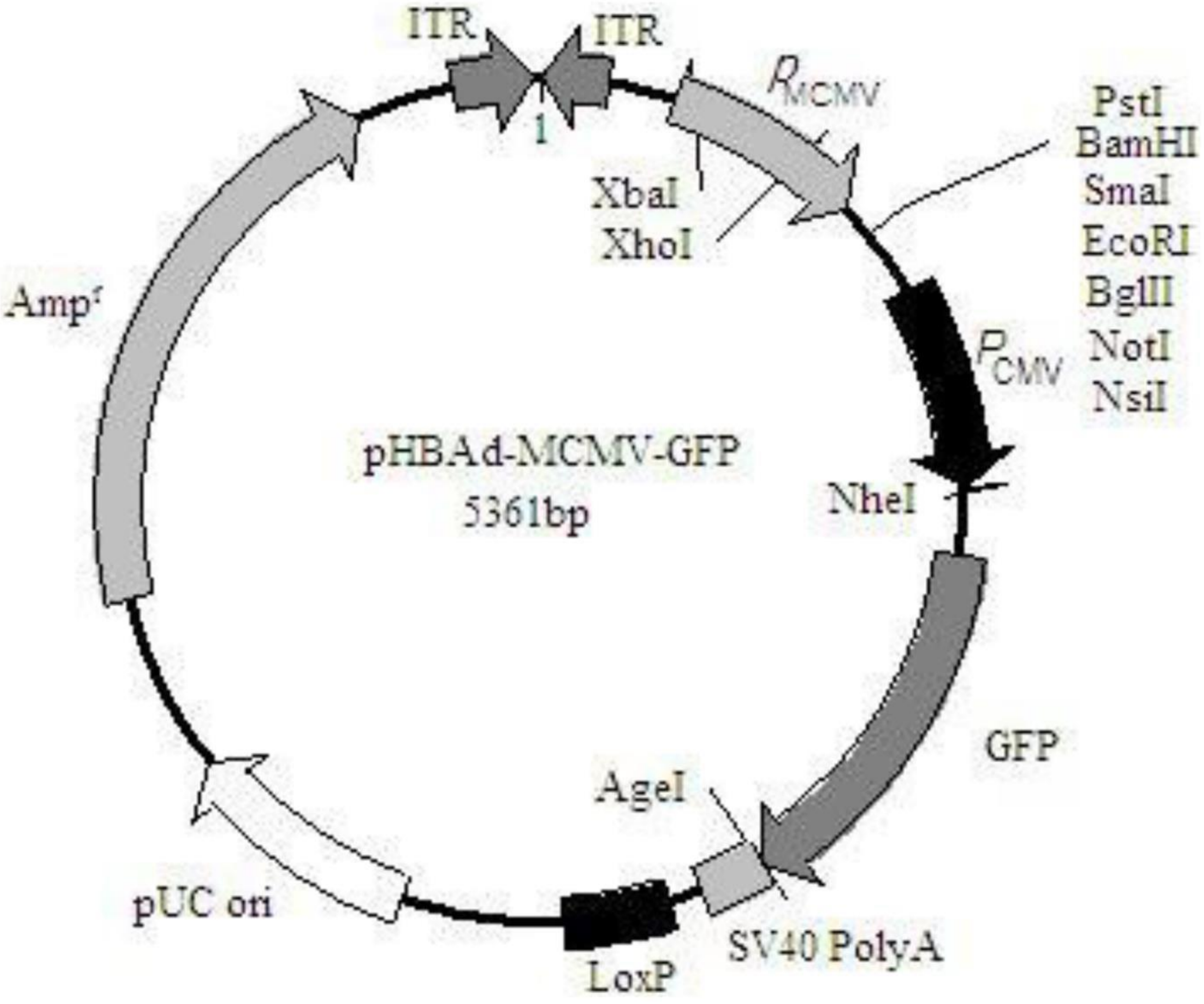

Supplementary Figure 9. Backbone map of adenoviral vector pHBAd-MCMV-GFP used in this study.

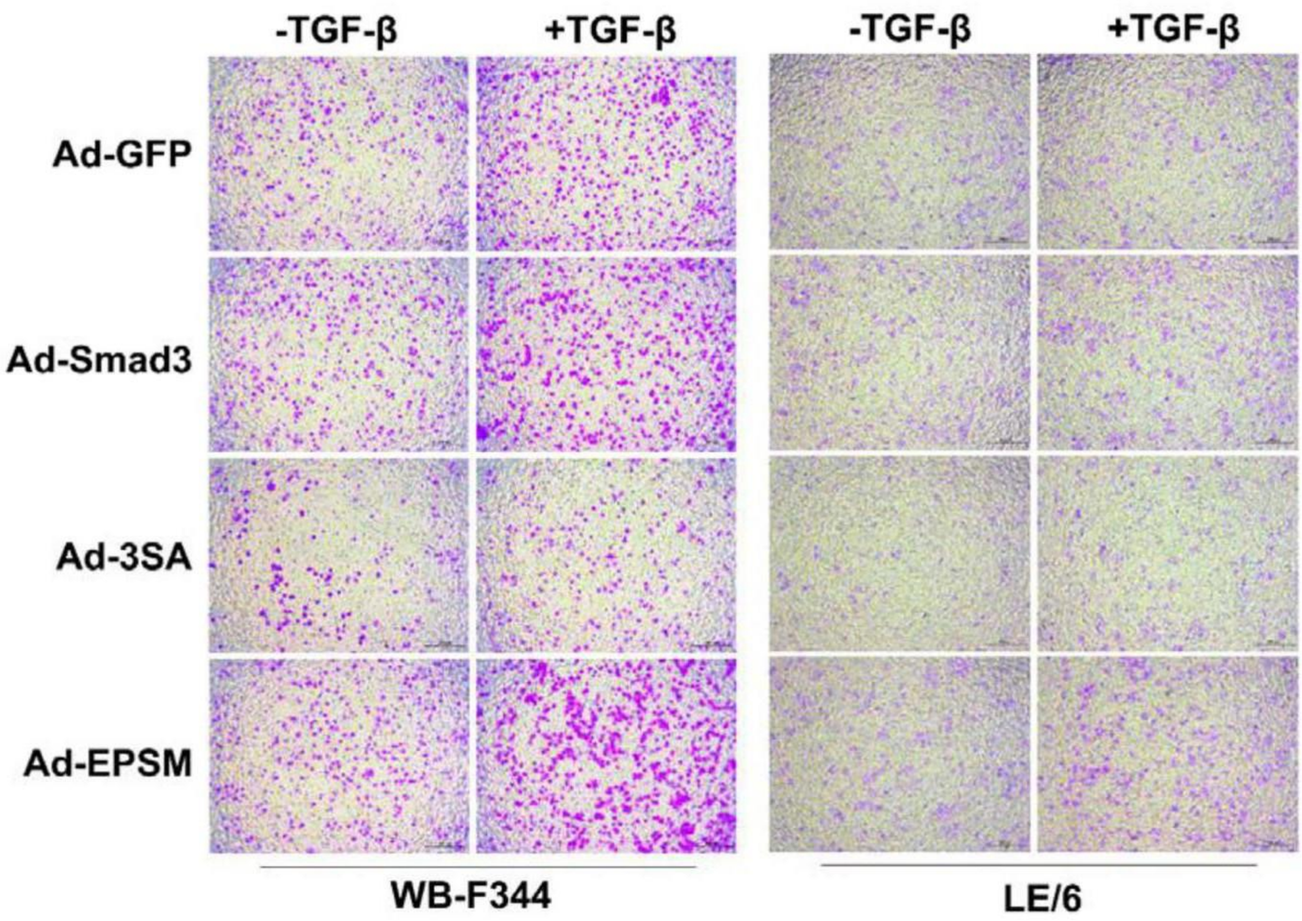

Supplementary Figure 10. Smad3 phosphorylation events affect TGF-β induced migration in LPCs, related to Figure 8. Representative images of cell motility assays as described in Figure 8.
