# Supplementary material for: TGF-β downstream of Smad3 and MAPK signaling antagonistically regulate the viability and partial epithelial–mesenchymal transition of liver progenitor cells
Source: Aging (Albany NY). 2024 Apr 5;16(7):6588–612. doi: 10.18632/aging.205725 (PMC11042936; doi:10.18632/aging.205725)
Supplement: Supplementary Tables [file aging-16-205725-s002.pdf]

SUPPLEMENTARY TABLES

Supplementary Table 1. Reagents used in this study.

Reagent	Source and identifier
Recombinant human TGF-β1 (carrier-free)	580706, BioLegend, San Diego, CA, USA
U0126-EtOH	S1102, Selleck, Houston, TX, USA
SP600125	1496, Tocris, Bristol, United Kingdom
SB203580 (Adezmapimod)	S1076, Selleck, Houston, TX, USA
Actin-Tracker Red-555	C2203S, Beyotime Institute of Biotechnology, Shanghai, China

Supplementary Table 2. Antibodies used in this study.

Antigens	Source and identifier	Application
Phospho-Smad2 (Ser465/467)	#3108, Cell Signaling Technology, Beverly, MA, USA	1:2000 for WB
Phospho-Smad3 (Ser423/425)	ab52903, Abcam, Cambridge, UK	1:1000 for WB
Phospho-Smad2 (Ser245/250/255)	#3104, Cell Signaling Technology, Beverly, MA, USA	1:1000 for WB
Phospho-Smad3 (Thr179)	ab74062, Abcam, Cambridge, UK	1:1000 for WB
Phospho-Smad3 (Ser213)	ab63403, Abcam, Cambridge, UK	1:1000 for WB
Phospho-Smad3 (Ser204)	ab63402, Abcam, Cambridge, UK	1:1000 for WB
Phospho-Smad3 (Ser208)	ab138659, Abcam, Cambridge, UK	1:1000 for WB
Smad2	ab40855, Abcam, Cambridge, UK	1:2000 for WB
Smad3	ab40854, Abcam, Cambridge, UK	1:2000 for WB
Smad4	ab40759, Abcam, Cambridge, UK	1:2000 for WB
E-cadherin	610181, BD Transduction Laboratories, San Jose, CA, USA	1:5000 for WB
Vimentin	#5741, Cell Signaling Technology, Beverly, MA, USA	1:20000 for WB
GAPDH	KC-5G4, KangChen Bio-tech, Shanghai, China	1:20000 for WB
Phospho-p44/42 MAPK (Erk1/2) (Thr202/Tyr204)	#4370, Cell Signaling Technology, Beverly, MA, USA	1:10000 for WB
p44/42 MAPK (Erk1/2)	#4695, Cell Signaling Technology, Beverly, MA, USA	1:10000 for WB
c-Jun Phospho (pS63) (JUN)	1527-1, Epitomics, Burlingame, CA, USA	1:1000 for WB
c-Jun (JUN)	1254-1, Epitomics, Burlingame, CA, USA	1:1000 for WB
Phospho-p38 MAPK (Thr180/Tyr182)	#4511, Cell Signaling Technology, Beverly, MA, USA	1:1000 for WB
p38 MAPK	#8690, Cell Signaling Technology, Beverly, MA, USA	1:1000 for WB
Horseradish peroxidase (HRP) conjugated anti-rabbit IgG	Jackson Immuno Research Laboratories, Inc. West Grove, PA, USA	1:5000 for WB
HRP conjugated anti-mouse IgG	Jackson Immuno Research Laboratories, Inc. West Grove, PA, USA	1:5000 for WB
